# The role of complementary and alternative medicine (CAM) in Germany – A focus group study of GPs

**DOI:** 10.1186/1472-6963-8-127

**Published:** 2008-06-12

**Authors:** Stefanie Joos, Berthold Musselmann, Antje Miksch, Thomas Rosemann, Joachim Szecsenyi

**Affiliations:** 1 Department of General Practice and Health Services Research, University Hospital Heidelberg, Voßstrasse 2, 69115 Heidelberg, Germany; 2 Practice of General Medicine, Academic Teaching Practice, University of Heidelberg, Hauptstrasse 120, 69168 Wiesloch, Germany

## Abstract

**Background:**

There has been a marked increase in the use of complementary and alternative medicine (CAM) in recent years worldwide. In Germany, apart from 'Heilpraktiker' (= state-licensed, non-medical CAM practitioners), some general practitioners (GPs) provide CAM in their practices. This paper aims to explore the attitudes of GPs about the role of CAM in Germany, in relation to the healthcare system, quality of care, medical education and research. Furthermore, experiences of GPs integrating CAM in their daily practice were explored.

**Methods:**

Using a qualitative methodological approach 3 focus groups with a convenience sample of 17 GPs were conducted. The discussions were transcribed verbatim and analysed using qualitative content analysis.

**Results:**

The majority of the participating GPs had integrated one or more CAM therapies into their every-day practice. Four key themes were identified based on the topics covered in the focus groups: the role of CAM within the German healthcare system, quality of care, education and research. Within the theme 'role of CAM within the healthcare system' there were five categories: integration of CAM, CAM in the Statutory Health Insurance, modernisation of the Statutory Health Insurance Act, individual healthcare services and 'Heilpraktiker'. Regarding quality of care there were two broad groups of GPs: those who thought patients would benefit from standardizing CAM and those who feared that quality control would interfere with the individual approach of CAM. The main issues identified relating to research and education were the need for the development of alternative research strategies and the low quality of existing CAM education respectively.

**Conclusion:**

The majority of the participating GPs considered CAM as a reasonable complementary approach within primary care. The study increased our understanding of GPs attitudes about the role of CAM within the German healthcare system and the use of 'Heilpraktiker' as a competing CAM-provider. It seems to be a need for increased funding for research, better education and remuneration by the Statutory Health Insurance in order to improve access to 'Integrative medicine' in Germany.

## Background

In Germany, the overall percentage of the population with experience of using Complementary and Alternative Medicine (CAM) increased from 52% in 1970 to 73% in 2002 [1]. A national representative survey shows that herbal medicine, exercise therapy and hydrotherapy are the most frequently used CAM modalities in Germany [2]. Besides these so-called classic naturopathic therapies, homeopathy, manual therapy and acupuncture are commonly used CAM therapies [2].

The extent to which CAM is practiced by physicians or non-medical therapists differs considerably among countries. In Germany, medical doctors can obtain a variety of additional qualifications relating to specific CAM therapies (e.g. chiropractic, homeopathy, naturopath*y*) regulated by the regional chamber of physicians. In 2005 the German federal medical chamber documented 46.000 qualifications in CAM relating to around 60.000 general practitioners. Among those qualifications the most prevalent were chiropractic and naturopathy [3] (Table 1). Recently, acupuncture was introduced as a 'new' CAM qualification (former 'acupuncture-certificate') accredited by local medical chambers. Although exact data are missing it is estimated that about 20.000 to 30.000 physicians are currently practising acupuncture in Germany. Furthermore, many physicians are providing CAM in their daily practice without having any CAM qualification or certificate [4].

**Table 1:** Qualifications in the field of CAM in the years 1993 and 2006

**Type of qualification**	**Doctors with qualification (n) 1993**	**Doctors with qualification (n) 2006**
**Naturopathy**	4.573	14.497
**Homeopathy**	1.905	6.073
**Chiropractic/Spinal Manipulation**	5.355	17.591
**Physical therapy**	1.991	6.146
**Balneology and medical climatology**	1.560	2.886

(Source: National Association of Social Health Insurance-accredited Physicians, Germany)

Only a small part of CAM is covered by the German statutory health insurance (SHI), namely physiotherapy, chiropractic, classic naturopathy (including phytotherapy) and to a lesser extent homeopathy, as well as acupuncture in patients with knee pain and lumbar pain. However, for reimbursement by the SHI, the therapist must hold the corresponding CAM-qualification. All remaining CAM modalities are not covered by SHI but have to be paid by the patients themselves as so-called 'IHS' (= **I**ndividual **H**ealthcare **S**ervices; in German '**I**ndividuelle **Ge**sundheits-**L**eistungen' = IGeL) or may be paid by private health insurances.

The SHI Modernization Act in 2004 (= GMG 2004) was a step backwards for CAM in Germany. At the beginning of January 2004 almost all non-prescription drugs were excluded from being reimbursed by the SHI [5], meaning that all homeopathics as well as phytotherapeutics (with the exception of mistletoe, St. John's wort, psyllium and ginkgo) were no longer reimbursable drugs. Furthermore, for the first time patients in general had to pay a consultation fee of 10 € per quarter to see their doctor.

In addition to physicians there are non-medical, state-licensed practitioners in Germany; the so-called 'Heilpraktiker', which were officially recognised by the 'Heilpraktiker'-law in 1935. A 'Heilpraktiker' has to pass an exam on basic medical knowledge and skills at a local public health office to obtain a state license. However, 'Heilpraktiker' are not obliged to undertake medical and/or CAM training. 'Heilpraktiker' only practice in the ambulatory sector and their services are not covered by the SHI but many private health insurances pay for 'Heilpraktiker'. Around 10% of patients have a private health insurance in Germany. Moreover, SHI patients increasingly take out an additional private insurance for CAM. Unlike physicians, 'Heilpraktiker' do not have to comply strictly with fee schedules and they are allowed to advertise their services to the public [6]. Any CAM therapy can be performed by a 'Heilpraktiker' as long as it is consistent with the 'Heilpraktiker'-law ('avert a danger to the health of the people') [7]. In general, 'Heilpraktiker' provide a great variety of CAM therapies. Complications are likely because they perform injections, for instance of homeopathic remedies, and other invasive procedures [8]. The number of 'Heilpraktiker' increased from 9.000 in 1993 to nearly 20.000 in 2007. So, with around 60.000 general practitioners (GPs) the ratio of 'Heilpraktiker' versus GPs is thought to be 1:3 at present [3]. The majority of 'Heilpraktiker' (>90%) are organized within 6 professional associations [9].

Little is known about the role of CAM within the German health care system and CAM is still a highly controversial issue among German doctors, generating considerable professional and public debate [10,11]. This paper explores the attitudes of GPs about the role of CAM in relation to the healthcare system and their everyday practice, quality of care, medical education and research in Germany. It also focuses upon the role of 'Heilpraktiker' within the German healthcare system.

All diagnosis and treatment modalities complementing 'conventional medicine' were included such as classical naturopathy (hydrotherapy, phytotherapy, kinesiotherapy, dietetics, physical and regulative therapy), acupuncture, chiropractic, homeopathy, anthroposophic medicine and neural therapy and also less well-known and used therapies such as bioresonance therapy and autohemotherapy.

## Methods

We chose a qualitative approach consisting of focus groups because little is known about perspectives and rationales of GPs regarding CAM in Germany. Focus groups are an established qualitative research method to collect information from particular groups e.g. professional groups [12]. Unlike single person interviews, focus groups are able to explore disagreements as well as defining consensus and to work with group interaction processes to uncover hidden attitudes. 5 to 8 participants are considered to be an optimal size for a focus group [13].

### Ethics approval

The ethics committee of the Heidelberg Medical School informed us that approval by an ethics committee was not necessary because the study does not involve patient data (email communication 17/08/05).

### Sampling

GPs were recruited during a symposium organized by the Department of General Practice and Health Services Research at the University Clinic of Heidelberg on the 8^th ^of October 2007[14]. An information leaflet in the congress folder describing the aim of the study was handed out during the symposium to around 100 participating GPs from the area of Heidelberg/Nordbaden. In addition, GPs participating in quality circles (= regular meetings of 5–12 GPs where issues and problems of the daily work are discussed in a structured way, guided by a qualified moderator as part of a continuous quality improvement process) to which authors are members were also invited to participate by post. The only inclusion criterion for participation in the focus groups was that GPs had to be working in a practice as a GP, albeit self-employed or employed. Participants did not necessarily have to practice CAM.

### Data collection

Discussions were moderated by two of the authors (SJ/BM). At the beginning of each of the 3 focus groups there was an introduction about the methodological apects of focus groups given by the moderator. Furthermore, aspects of CAM terminology were presented on 3 power point slides to avoid discussions about 'terms'. Each focus group began with a round of introductions including information about age, location and structure of practice, years of work in practice, CAM qualification and focus of interest. In the course of each focus group the following central questions were presented by the moderator, each on a separate slide, to structure discussions:

• How do you assess the current role of CAM in SHI?

• Did the SHI Modernization Act (GMG 2004) make any changes to your every-day practice?

• What benefits or risks do you see in Individual Healthcare Services (IHS)?

• What do you think about 'Heilpraktiker'?

• What do you think about quality in CAM?

• What do think about CAM education?

• What do you think about research in CAM and what research topic would you personally be interested in?

The moderator ensured that each aspect of these questions was explained sufficiently, so that no questions or misunderstandings remained. During discussions the moderator ensured that contributions to the discussions were distributed more or less evenly among participants, i.e. where necessary the moderator might have asked some of the participants directly for his/her opinion.

### Data analysis

The interviews were recorded digitally and fully transcribed. Each GP was labelled with a number. GP1 – GP 7 were particpants of focus group 1, GP 8 – GP12 were participants of focus group 2 and GP 13 – GP 17 were participants of focus group 3. Trancripts were analysed according to the qualitative content analysis of Mayring [15]. ATLAS.ti software was used for coding, text searching and developing network views [16].

Four key themes were identified based on the topics covered in the focus groups: role of CAM within the German healthcare system, quality of care, education and research. All transcripts were read independently by two of the research team (SJ, BM) and a preliminary coding frame constructed with key themes and categories identified using an open coding method until a consensus was achieved. At regular research team meetings codes were merged within ATLAS.ti. The quotations cited here were translated by SJ from German into English and cross-checked by BM.

## Results

17 GPs were recruited for participation in the focus groups. 12 GPs were recruited among congress participants whereas 5 GPs were recruited from quality circles of the authors. 3 of the GPs had academic links to the Department of General Practice and Health Services Research, Heidelberg University in addition to working in practice. Table 2 shows the main characteristics of the participating GPs. The majority of participants practised some form of CAM, with 4 GPs stating in the round of introductions that they did not practice CAM.

**Table 2:** Characteristics of participating GPs

**Gender:**	f: 9
	m: 8
**Years of work in practice**	7,6 years
	(min 0,5 J.; max. 15 J)
**Structure of practice:**	single practice: 7
	group practice: 8
	Practice-sharing: 2
**Location of practice:**	city: 12
	rural: 5
**Qualifications of GPs:**	naturopathy:14
	chiropractic:4
	homeopathy: 5
	balneology: 1
	(acupuncture certificate: 4)

Each focus group lasted about three hours. The content analysis of the focus group transcripts resulted in four deductively derived themes (based on topics prompted for within the groups) containing several inductively derived categories (see Table 3).

**Table 3:** Key themes and categories

**Key themes**	**Categories**
**role of CAM within healthcare system**	• integration of CAM
	• CAM in SHI
	• modernisation law (GMG 2004)
	• individual healthcare services (IHS)
	• 'Heilpraktiker'
**quality**	• difficulties of quality control
	• quality criteria
	• standards/guidelines
	• quality control as instrument for rationalisation
**education**	• CAM should be included in undergraduate education
	• Parallel continued medical education in CAM and conventional medicine
	• quality of courses needs improvement
	• standardised formats
**research**	• individual approach of CAM
	• research methodology
	• relevance for practice

### Role of CAM within the German healthcare system

The key theme 'role of CAM within healthcare system' was discussed very extensively. The categories with their associated codes are summarized and displayed in Table 4.

**Table 4:** Categories and codes for the key theme 'Role of CAM within healthcare system'

**Categories**	**Codes**
**integration of CAM**	• integration as continuum
	• evidence as criterion for integration
	• patient as criterion for integration
	• role of CAM in ‚preventive medicine’
**CAM in SHI**	• CAM within the catalogue of benefits of SHI?
	• insufficient remuneration for ‚time’/narrative-based medicine in the present healthcare system
	• reduction of overall healthcare costs by CAM
	• lack of linkage among existing offers
**modernisation law (GMG 2004)**	• negative consequences for physicians
	• negative consequences for patients
	• positive consequences
**Individual Health Services (IHS)**	• risks of IHS
	• commercial role of GP endangers doctor-patient relationship
	• transparent communication about IHS
	• advantages of IHS
**'Heilpraktiker'**	• *see Figure *1*and Figure *2*for codes*

#### Integration of CAM

Most GPs reported that they have integrated one or more CAM therapies into their daily practice. GPs described their 'integrated' every-day practice as a continuum of possible medical approaches ranging between 'conventional therapies' and 'natural therapies'.

Existing evidence and patient preference are the main criteria used by GPs in deciding whether to apply CAM or conventional therapy. Important characteristics to be considered, therefore, are the presenting complaint as well as the experiences and expectations of the patient.

GPs criticized the lack of linkage among existing CAM-offers covered by SHI such as 'back care courses' or 'yoga courses' provided by public gyms. GPs complain about the fact that patients often make use of those courses unbeknown to their doctors. The majority of GPs considered that, to optimize benefit for patients, such courses should be integrated in a comprehensive therapeutic plan. In this regard, a key role for GPs that practice CAM is to link and integrate existing services.

'So, when I look at this brochure of [name of a public gym], I see back care courses, courses for dietetic treatment – who pays? Statutory health insurance....then I think.... [a colleague interrupted: outsourced so to speak]...yes, well 'past'-sourced upon my regulative therapy concept.' (GP 13)

Overall, most GPs regarded integration of CAM as an opportunity for providing health care with a lower level of side effects and a more preventive approach while also actively involving patients in decision-making resulting in better patient health outcomes.

#### Statutory health insurance (SHI)

In all focus groups GPs complained that there is not sufficient payment for the time spent listening and talking to patients. GPs emphasised that, since time with the patient is the basis for many CAM therapies, better remuneration of this 'time' by SHI would enable enhanced provision of CAM.

In this context GPs critisized the recent regulations concerning SHI remuneration for acupuncture, which consists of 20–25 € per acupuncture session. GPs complained that this amount does not cover the time needed for a comprehensive Chinese diagnosis and/or potential details emerging in the course of treatment. Therefore, some GPs refused to provide acupuncture under these conditions.

Extended inclusion of CAM within the catalogue of benefits of the SHI was a matter of considerable debate among the GPs. Some regarded evidence-based medicine as a suitable tool to decide which CAM therapy – as well as conventional therapy – should be covered by SHI. It was suggested that generating evidence in CAM is problematic due to a lack of research funding and perceived barriers to conducting CAM research at Universities. As such, some GPs wondered what should be done 'in the meantime'.

'In case one would try to restructure Statutory Health Insurance covered medicine, it would be necessary to separate much waste: useless meniscus surgeries and intracardiac catheters in heaps performed without sufficient diagnosis ...... However, nobody would join it. That's the problem. But there would be enough money.' (GP 5)

Some GPs postulated that an increased use of CAM would decrease overall costs in the healthcare system, because lower direct costs (fewer and cheaper drugs) and lower indirect costs would result from increased patient empowerment as a result of using CAM.

#### SHI Modernization Act (GMG 2004)

GPs repeatedly described that practices that provide CAM have lost patients since the introduction of the GMG in 2004. Suggested reasons included the ending of reimbursement for phytotherapeutic and homeopathic drugs and the newly introduced consultation fee. The consultation fee is particularly important as an explanation for the loss of patients in practices where the doctor serves as 'CAM-deluxe-doctor', visited in addition to the 'normal' GP.

Furthermore, GPs observed that the introduction of the GMG 2004 had meant that some patients with acute symptoms delayed their presentation. This would make using a CAM approach more difficult. On the other hand, serious symptoms may be identified (too) late or diseases may be protracted. Further negative consequences of the GMG 2004 were seen in terms of poorer patients stopping taking phytotherapeutic drugs. However, several GPs reported that patients continued treatment with 'phytos' after a short break paying for them out of their own pocket.

'There are some patients saying: 'No, that is too expensive for me', for example with these ginkgo preparations ('My pension is not enough'), but most patients were saying: that was helping me so far, I would like to continue with this. (GP 8)

Some GPs reported that, with the introduction of the GMG 2004, they differentiated more strictly between medical services covered by SHI and any further medical services, which patients apparently accept. Moreover, it was suggested by the GPs that the GMG 2004 had the paradoxical side effect that patients now pay more willingly for individual healthcare services (IHS) because they have realized that SHI does not pay for CAM and, therefore, that the doctor does not get paid for the time, he/she spends providing CAM.

'Increasingly, we offer individual healthcare services. In the past we have injected vitamins ... without taking money. Since 2004 we charge 5 € per injection and I must say this is accepted by patients more and more. The problem, I think, is more on my side as a doctor. For example, we have a homeopathic colleague....and when we cover his practice, patients come with their homeopathics and ask already when registering: 'What do I have to pay?' This is completely normal.' (GP 10)

A few GPs mentioned that the GMG 2004 had resulted in more relaxed consultations. This might be explained by the fact that phytotherapeutics and homeopathics no longer burden the GP's sectoral budget for drugs. So, decision-making lies more with the patient.

'And that is why I am more relaxed now. In case a patient wants to have something 'natural', then he/she has to pay for it. Certainly, I give advice but I am no longer stressed by patients saying: He prescribes this but not that one...and so on. This is beneficial.' (GP 2)

#### Individual Healthcare Services (IHS)

There was disagreement among GPs as to whether CAM should be provided as IHS. Most of the participating GPs saw the risk that patients might be exploited by doctors offering unproven, possibly harmful therapies. This situation gets more dramatic by expanding the spectrum of indications on the basis of dubious diagnoses with the aid of technical equipment such as kirlian photograpy. However, this risk was also seen for IHS in conventional medicine and, therefore, was regarded as a problem for out-patient healthcare in Germany.

'It starts with dubious equipment for a few thousand Euros: ozone-therapy, colon-hydrotherapy or bioresonance-therapy. At the same time an amortisation schedule sets a plan how many patients per quartile have to be diagnosed or treated, that the equipment turns out to be profitable.' (GP 5)

A negative consequence of IHS was seen in the upcoming commercial role of the GP which might endanger the trustful doctor-patient relationship. Most GPs stated that they are not willing to accept this role.

'...and when I say that CAM should have more importance in our healthcare system – beyond IHS – then, because I do not want to be a businessman putting my vitamins and dietary supplements on the desk and opening a branch on the name of my wife.' (GP 13)

There was consensus among the GPs about communication regarding IHS: all services should be made available for all patients. Furthermore, IHS should only be provided after a thorough and honest discussion with the patient in advance. Most GPs were of the opinion that for offering a certain CAM therapy one's belief in the efficacy of this therapy is an essential precondition.

In cases where the decision in favour of CAM is made on demand or in agreement with the patient and not imposed on the patient by the doctor, some GPs see an advantage in IHS. They suggest that the fact to pay extra for a specific therapy has a positive impact on patients' expectations which may increase the overall benefit of the treatment.

'A chance of IHS may be, that, yes, the meaning of something you have paid for is different from something you get for free. And this, maybe, is assessed or realized different by patients...' (GP 16)

#### 'Heilpraktiker'

**Figure 1:**
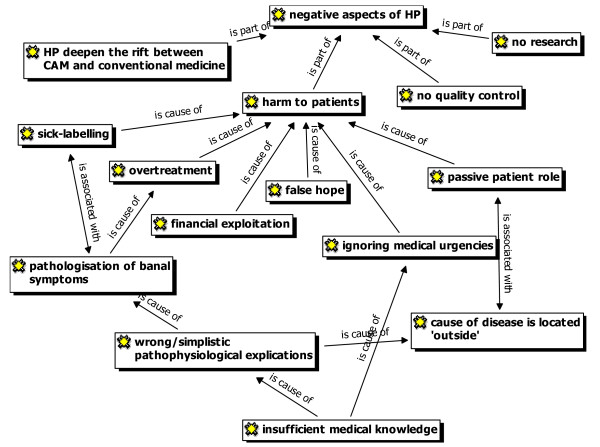
Negative arguments with regard to Heilpraktiker (HP) raised by GPs (ATLAS.ti network view).

**Figure 2:**
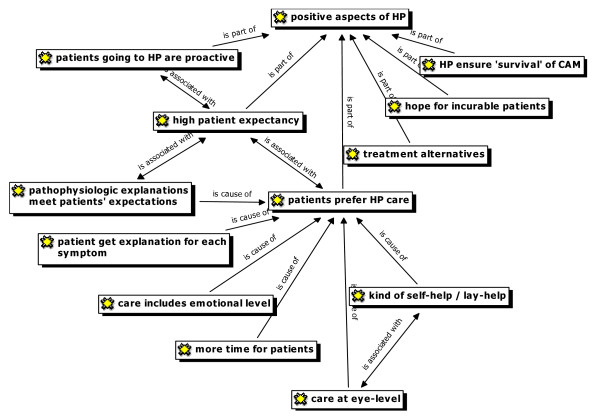
Positive arguments with regard to Heilpraktiker (HP) raised by GPs (ATLAS.ti network view).

Opinions towards 'Heilpraktiker' differed widely among participating GPs. The main positive and negative arguments of the discussions are presented in Figure 1 and Figure 2.

Most GPs worried that the qualifications of 'Heilpraktiker' may not fully enable such practitioners to identify certain symptoms and diseases or to assess the urgency in certain medical conditions. Furthermore, GPs stated that 'Heilpraktiker' tend to provide patients with simple, one-dimensional explanations for their symptoms. This leads to the development of simplified and, partly, wrong medical theories in patients.

'Sometimes you hear remarks of patients about correlations and therapies in conventional medicine: thyroid hormones weaken the thyroid gland. Those wrong or simplified medical theories and absurd ideas circulate among the total population.' (GP 5)

Another negative aspect mentioned by the GPs was that 'Heilpraktiker' might encourage a pathologization process of medical complaints providing simplified, often premature explanations. On the one hand those explanations often label the patients with irreversible diagnoses. On the other hand, some GPs supposed that a therapeutic demand is created leading in extreme cases (e.g. in patients with unfavourable or fatal prognosis) to addictions and the financial exploitation of a patient.

'So, I think, pilgrimage is a good word for this. I have a patient, who drives hundreds of kilometres to a 'Heilpraktiker' just putting stones on her belly. She goes there once in a quarter and pays 800. – €. She says she would need that.' (GP 8)

Another critisicm expressed by some GPs is the fact that 'Heilpraktiker' usually avoid giving patients responsibility for her/his condition but blame an 'enemy from outside' such as intestinal mycosis which is a typical 'Heilpraktiker' – diagnosis for lots of complaints.

'Giving patients co-responsibility for their situation... patients do not like this. And the 'Heilpraktiker' says: There is an enemy in your body – we will fight this enemy. This relieves. Not you have to do anything. You only have to let it done.' (GP 11)

Alternatively, it was recognised that 'Heilpraktiker' spend more time with patients and are seen to be on 'folks side' and represent, therefore, a kind of self-help or lay-help. Possibly, patients consulting 'Heilpraktiker' are looking for help on an 'eye-to-eye level' and, – some GPs suggest – might feel better off with 'Heilpraktiker' than with doctors resulting in improved adherence to treatment.

'But the general idea is not so bad to educate a kind of lay-help for minor complaints. This could save the professional system work.' (GP 5)

Most GPs supposed that the popularity of 'Heilpraktiker' is linked to patients' low level of trust in regular healthcare. GPs presume that some 'Heilpraktiker' take advantage of this situation and try to generate a special 'esoteric' aura to attract people.

In Germany, many patients can still not differentiate between physicians practising CAM and 'Heilpraktiker'. Therefore, GPs regarded a public-oriented demarcation from 'Heilpraktiker' as important. In this regard, some GPs criticised the lack of a single affiliation among doctors practising CAM, including a strong professional organisation representing this group of doctors.

'Often I hear that from my patients: Are you a 'Heilpraktiker'? Either the image of CAM-practising physicians is so bad or the healthcare system is so intransparent to our patients. There is still a lot to be done to sharpen our profile.' (GP 14)

In summary, 'Heilpraktiker' are partly seen as competitors and partly as co-workers filling the gap by providing services that focus on humanity, time and empathising with patients who often complain about the contemporary medical system .

### Quality

The definition and application of quality standards in the field of CAM was a controversial issue among participating GPs. There were two broad groups of GPs: those who thought that patients would benefit from standardizing CAM and those who feared that quality control would have negative effects for patients affecting the more subjective, emotional aspects of the therapeutic/healing process. Nevertheless, both groups considered the development of quality criteria would be useful to limit uncontrolled growth of 'medical grey areas' underpinned by only limited 'evidence-base'. Quality control was considered particularly important by GPs for CAM therapies covered by the SHI.

'I guess, seriousness and limitation of this 'creating-his-own-market-mentality' can only be obtained by evaluating CAM, standardizing CAM and implementing CAM in medical training and eduction on a level as high as possible.' (GP 8)

Across all focus groups there was a lively discussion about the extent to which pre-defined quality criteria can be developed relating to a patient's bio-psycho-social context, opinions and expectations. However, the more sceptical group of GPs worried that those CAM therapies involving a complex intervention would lose a part of their therapeutic efficacy and the core element of individuality by standardization.

'It will be possible for certain CAM therapies. But for therapies where doctor-patient relationship, expectancies, time and so on play a major role – as for example in psychosomatic medicine – it becomes more and more difficult.' (GP 9)

Patients' perceived benefit of a therapeutical approach was seen as an essential quality criterion by the GPs. Therefore, adequate communication before, during and after completion of a therapeutic (CAM)-procedure is important.

'First, there must be a definition of the aspired result. What do I expect? What does the patient expect? What does he need? What do I think does he need.' (GP 3)

'But this means also quality: trying to find out what patients expect. When I have the impression that somebody is not ready at all for a certain therapy then I will certainly not force him. I will look for another vehicle.' (GP 8)

Furthermore, most GPs regard 'time' as one of the most important quality indicators for a successful CAM therapy. Some GPs mentioned that it would be important to incorporate physician-factors within quality assessment of CAM because effects of many CAM therapies seem to be 'therapist-dependent'. However, since this concerns 'soft skills' (e.g. ability of empathy, ability to communicate) rather than 'technical skills', this was seen as problematic by most GPs. Another problematic but important indicator was the 'authenticity' of the physician and their own belief in a therapy. Some GPs supposed that patients know immedicately whether the doctor him/herself is not convinced of a certain therapy. Thus, the 'authenticity' of the therapist was perceived to have a substantial impact.

Furthermore, the 'primum non nocere-principle' (= 'first, do not harm') was mentioned as a basic principle of quality, in particular, when administering substances to a patient such as phytotherapy or Chinese drugs. In the view of the GPs, only proven preparations should be implemented. Altogether, GPs seemed to be sceptical about the desirability of developing quality criteria, worrying that essential elements of CAM might be missed. Some GPs were also suspicious that quality criteria would be used for cost containment.

### Education

There were only a few comments on the role of CAM at medical schools. Most of the GPs considered that CAM should be integrated in undergraduate education. For post-graduate education, GPs considered a parallel continuing medical education in both conventional and CAM topics to be important, in order to obtain and maintain a broad medical perspective. Overall, GPs seemed to be unsatisfied with the quality of CAM courses. Predominantly, the courses were seen to be too expensive and have poor content, poor presentation and a lack of structure. Moroever, the content of courses was dependent upon the provider of the courses. Several GPs reported that some of the courses resemble marketing events with referees often showing self-importance and overstatement concerning their own therapy.

*'Anyway, this referent was so arrogant...HIS therapy cures everything: myocardial infarction, asthma etc.... Such a performance is not acceptable.' (GP 8)*.

GPs recommended that courses should be based on formats that focused on practice-oriented content in small groups or quality circles combining practice-oriented learning and independent, well prepared evidence-based information.

### Research

Concerning the issue research GPs differentiated between CAM therapies with a rather individualised approach (homeopathy, TCM) and others (phytotherapy, autohemotherapy).

For the latter GPs estimated an evidence-based research approach as possible and reasonable. Particularly, research in phytotherapy was demanded by GPs since – from their point of view – there is huge patient demand for phytotherapeutics.

In contrast, regarding CAM therapies with an individualised approach, the majority of GPs doubted that current research methodology will lead to 'evidence'. To evaluate those CAM therapies, development and adoption of alternative research strategies was demanded. Those alternative strategies should incorporate individualised patient-centred outcome measures. Furthermore, studies comparing conventional routine care' versus complementary routine care' were suggested by the GPs. GPs emphasised that results of research influence them regarding their choice and exertion of certain CAM therapies.

'Even though not all publications are 100% close to reality, I feel more comfortable with my individual therapeutic decisions when I know that there are at least a few positive studies.' (GP 1)

## Discussion

This paper explores the attitudes and experiences of German GPs about the role of CAM in the German healthcare system. Participating GPs range between the ‚typical country doctor’ and the 'CAM-deluxe doctor' who typically practices in city areas and is visited in addition to the 'normal' GP. The majority of the participating GPs used one or more CAM therapies in every-day practice (beyond dietetics, regulative therapy and physical therapy).

### 'Time' as key factor

An important finding of the study relates to the fact that the time required to provide CAM is not valued or remunerated appropriately. GPs emphasised that the basis for most CAM therapies, as well as for good primary care, is a diagnostic/therapeutic approach which considers the bio-psycho-social context of a patient. Studies have shown that sufficient time in the consultation as well as making a patient feel valued and understood as an individual by the doctor are aspects closely linked with patients' perceptions of good consultation quality [17]. Furthermore, there is evidence that the empathy of practitioners, as perceived by patients, has a direct impact on patient enablement and health outcome [18,19]. Consistently, GPs in our focus groups emphasised that those aspects are not adequately considered in the German healthcare system. Therefore, changing remuneration to reflect the 'time' necessary to provide CAM was seen as key factor to improve the situation in Germany and an essential precondition for applying CAM in practice. A further important aspect in this connection is the dissatisfaction of Germans with their healthcare system [20]. In a study comparing three European countries people in Germany show less trust in healthcare, while people in England and Wales have the highest trust levels [21]. According to the literature a longer relationship with the physician, doctor's communication skills and doctor-patient interaction are determinants for patients trusting in healthcare [22,23]. The lack of time spent by doctors with individual patients in Germany might be one explaining factor for this low level of trust in German patients.

### Non-medical CAM practitioners (Heilpraktiker)

A lack of trust may cause patients to look for help on the ‚grey area’ of the health market. Thus, the great demand for 'Heilpraktiker' as non-medical CAM practitioners in Germany was attributed by GPs to patients' low level of trust in regular healthcare. Since, in particular chronically ill patients show low levels of trust and their number may further increase in future, health policy makers should, therefore, be alert to the quality of non-regular healthcare providers such as 'Heilpraktiker'.

As a basic principle, the majority of GPs advocated that only health services provided by physicians, and not by 'Heilpraktiker', should be reimbursed by health insurances (also private ones). Furthermore, education and licensing, but also issues such as fees and advertizing promoting 'Heilpraktiker' compared to physicians, should be reconsidered and re-organized. It is suggested that changes in remuneration which encourage doctors to spend more time with patients would improve the doctor-patient interaction and, consequently improve trust in regular healthcare. This might also result in a decreased demand for non-regular healthcare such as 'Heilpraktiker'.

### SHI Modernization Act 2004

The SHI Modernization Act had made little change to those GPs practising CAM. Some reported temporary decreases in consultations and increased prescriptions of antibiotics. These subjective impressions of the GPs are confirmed by survey data: immediately after the introduction of the consultation fee in January 2004 the number of consultations decreased by 8% to rise again above baseline values in the late 2005 [24]. At the beginning of 2004 increased prescriptions for antibiotics and antitussives – possibly instead of phytotherapeutics – were observed in a study evaluating patients with acute cough [25]. Nevertheless, the SHI Modernization Act seemed not to have left behind a strong influence on 'CAM care'. However, the impact on research, e.g. in the area of phytomedicine was substantial. Due to decreasing turnovers of phytotherapeutic drugs pharmaceutical companies invested less in producing phytotherapeutics – at least temporarily in 2004 and 2005.

### Individual Healthcare Services (IHS)

Most GPs vehemently rejected a role for themselves as businessman/businesswoman for IHS. They were very critical of colleagues offering dubious therapies and charging a lot of money. However, CAM is only a small part of this 'second health market' of IHS. In particular, in the diagnostic field IHS increased (e.g. fitness check-ups, tonometry) whereas in the therapeutic field – including CAM – IHS only moderately increased [26]. In connection with IHS, GPs regarded as positive that patients specifically search and pay for CAM might have higher expectations on the effect of a therapy. Indeed, there is evidence that high patient expectations may result in better outcomes [27,28]

### Costs

Repeatedly within the focus group discussions the opinion was advanced that the overall costs of the healthcare system could be reduced by integration of CAM. Until now, this hypothesis could not be explicitely confirmed by studies [29,30]. However, the study of Busato et al provided evidence of another composition of costs in CAM-physicians indicating a more patient-centred care [30]. Thus, CAM-physicans were more expensive regarding consultations whereas expenditures for drugs were less compared to Non-CAM-physicians [30]. Another longterm observational study showed a modest reduction of sick leave in chronically ill patients receiving a multimodal CAM therapy [31].

### Quality

Regarding quality assessment two main issues were discussed: the need to define quality and the concern about losing the key components of CAM while doing so. As comprehensive quality criteria for CAM GPs mentioned physician-factors such as authenticity and empathy. Indeed, there is plenty of evidence that physician-factors, in particular psychosocial factors rather than 'technical' skills, have a significant impact on the outcome of a therapy [32-34]. For example, regular Balint training may enhance psychosocial skills in GPs providing CAM.

However, GPs raised the question as to how to represent psychosocial physician factors within quality assessment. Existing measures include the 'Consultation and Relational Empathy' (CARE) measure [35] or the 'Medical Interview Satisfaction Scale' (MISS-29) measure [36] developed to assess patient satisfaction with individual doctor-patient consultations. Both measures were developed for general practice.

### Research

The discussions on 'research' reflected the incongruity between the individual patient approach of CAM and the application of modern research methodologies represented by the RCT-design. Developing study designs that are more appropriate for CAM was regarded as challenge for the future, which should take into account the concept of the different CAM procedures and personal experiences of therapists and patients. However, regarding most CAM therapies as 'complex interventions' there is an existing research methodology which can be transferred to CAM research [37,38].

Networking involving universities and professional associations was suggested by the GPs to bring forward CAM research. A start has been made with networks such as the FORUM (Forum of Academic CAM research Groups) comprising colleagues from Germany, Switzerland and Austria [39]. Furthermore, cross-national initiatives for inclusion of CAM into the 7^th ^EU Research Framework Programme and the following common research proposals attest to improved networking on an European level [40].

### Education

Overall discussions about educational issues were less detailed but recommended that CAM should be included in undergraduate education. Existing postgraduate courses were widely critisized as insufficiently evidence-based and too expensive. Improvement in the quality of professional education including formats combining practice-oriented learning and well prepared evidence-based information in small groups or quality circles was requested by GPs.

### Limitations of the study

Because the sample was a convenience sample it is possible that the GPs that did not respond to the invitation were less enthusiastic about CAM and, therefore, may have different attitudes on the role of CAM within the German healthcare system. While there was at least one GP in each focus group who did not practise CAM, the majority of the sample practised some form of CAM and therefore might have expressed more positive views than CAM 'sceptics'. However, according to a recent survey around 60% of German GPs practice some type of CAM (own data; publication in preparation).Therefore, findings derived from this sample might reflect experiences and perspectives not only of a minority of German GPs. The question remains as to how transferable these findings are likely to be to other countries and health care systems respectively. It seems likely that the findings may be transferable to settings in other countries where GPs have integrated some form of CAM within their practice. However, there might be differences regarding issues of SHI and individual health services because these issues are tightly linked with political, country-specific regulations. Although the construct of 'Heilpraktiker' is unique in Germany, non-medical therapists such as chiropractors or acupuncturists may have similar functions in other countries. It might be interesting to explore similarities and differences between GPs attitudes on CAM and on non-medical CAM practitioners across different countries in future studies.

As it is the nature of qualitative research, the analysis of the texts may be influenced by the authors, who are both involved in providing and teaching CAM (SJ, BM). By means of continuing discussions with all authors we have tried to minimize those influences. Because we reached saturation in the analysis of the key themes after 3 focus groups we stopped recruiting GPs. Due to the differences in healthcare systems in Germany and other countries we have tried to explain all relevant facts in detail [41]. Nevertheless, in the present article there might remain some ambiguity caused by differences in healthcare systems.

## Conclusion

The majority of the participating GPs considered CAM as a reasonable complementary approach within primary care. However, higher remuneration of the 'time' requested to provide CAM is the main prerequisite for realizing 'integrative medicine' in Germany. CAM should be implemented on the basis of evidence considering patient's individual demands, values and socio-economic background as well as on the expertise of the individual doctor. However, comprehensive funding is a precondition to generate evidence and to further evaluate the cultural, social, economic and political aspects of CAM. In future studies, the quality of the doctor-patient-relationship and differences in patients' outcomes in doctors applying CAM compared to Non-CAM-physicians should be assessed.

## Competing interests

The authors declare that they have no competing interests.

## Authors' contributions

SJ and BM conceived the study, conducted and analysed the focus groups. SJ drafted the manuscript. AM, TR and JS were involved in the study design and made contributions to the manuscript. All authors read and approved the final manuscript.

## Pre-publication history

The pre-publication history for this paper can be accessed here:


